# 

**Published:** 1916-09-09

**Authors:** 


### HOSPITAL AND INSTITUTIONAL RESULTS IN 1915.

**Essex County Hospital, Colchester.**—The report has on the cover an interior view of one of the Netley huts for wounded soldiers, plans of which are given inside. The hospital has been able to provide, in these huts and elsewhere, accommodation for about 150 men without any curtailment of the work for civilian patients. Total income (including £161 legacies and £2,581 for emergency buildings, etc.), £9,160. Total expenditure (including £2,978 extraordinary), £10,153.

**Royal Southern Hospital, Liverpool.**—The supporters of the hospital are invited to give donations in memory of those who have fallen in the war; these gifts will be recorded on special pages in the annual report. The Queen has graciously agreed that the title of the hospital shall be changed from the New Southern Hospital to the Royal Southern Hospital. Total income (including £7,916 legacies), £20,081. Total expenditure, £15,883.

**Walsall and District Hospital.**—The cover of the report bears an attractive photograph of the Florence Nightingale Memorial. The total of in-patients treated was 1,001—a record—and the number of out-patients was greater than in 1914. Thirty beds are used by the wounded. Total ordinary income, £4,811; extraordinary, £1,146; total expenditure, £5,928. The hospital has been informed of the bequest of £10,000 by the late Mr. James Mason, of Melbourne.

**Cookridge Convalescent Hospital, Leeds.**—The hospital is used entirely by the military authorities, but a branch for the use of women only has, by the kindness of Sir Hickman Bacon, been opened at Westwood Hall. Total income (including £2,300 War Office contribution), £4,212; total expenditure, £3,929.

**The Royal National Sanatorium, Bournemouth.**—506 patients were admitted in 1915. The committee are considering a scheme for removing the work to a farm colony. Total income (including £1,396 legacies), £8,653; total expenditure, £6,036.

**The United Kingdom Band of Hope Union.**—The accounts presented are from April 1 to March 31. The estimated membership is 3,393,383, but the method of enumeration is far from reliable.

### MORE GALLANT R.A.M.C. OFFICERS.

Since the announcement of the awards for gallantry and devotion to duty in the field referred to in The Hospital of August 26 (p. 498) a further list of awards has been issued, which includes the names of a number of medical officers. The D.S.O. has been awarded to Temporary Lieutenant H. L. Glyn Hughes, R.A.M.C., and to Temporary Captain H. H. Robinson, R.A.M.C., both of whom performed deeds of great bravery. The Military Cross has been awarded to the following officers: Captain W. Barclay, M.B., R.A.M.C.; Temporary Lieutenant J. R. Boyd, M.D., R.A.M.C.; Temporary Captain M. C. Burke, M.D., R.A.M.C.; Temporary Lieutenant C. B. Davies, R.A.M.C.; Temporary Captain J. C. Dunn, R.A.M.C.; Temporary Captain E. H. Griffin, M.D., R.A.M.C.; Captain H. A. Harbison, R.A.M.C.; Captain W. Johnson, M.D., R.A.M.C.; Temporary Captain J. R. Kemp, R.A.M.C.; Captain G. H. H. Manfield, R.A.M.C.; Temporary Captain V. C. Martyn, R.A.M.C.; Temporary Captain J. B. Orr, R.A.M.C.; Temporary Captain D. Pottinger, M.B., R.A.M.C.; Temporary Captain C. W. Roe, R.A.M.C.; Captain J. W. Scott, M.D., R.A.M.C.; Temporary Captain J. H. Tomlinson, R.A.M.C.; and Captain F. McIntyre, A.M.C. The records show that all these officers acted with conspicuous bravery under circumstances of the greatest danger.

### The Book Market.

LIST OF NEW BOOKS RECEIVED FROM THE FOLLOWING PUBLISHERS:—

The Panini Office, Allahabad: "The Dietetic Treatment of Diabetes." By Major B. D. Basu, I.M.S.

Longmans, Green and Co.: "Gray's Anatomy. Descriptive and Applied." 19th Edition. 32s. net. "Notes on the Causation of Cancer." By the Hon. Rollo Russell. 3s. 6d. net.

H. K. Lewis and Co., Ltd.: "The Mentally Defective Child." By Meredith Young, M.D. 3s. 6d. net.

Macmillan and Co.: "Discovery: The Spirit and Service of Science." By R. A. Gregory. 5s. net.

G. P. Putnam's Sons: "The People who Run: Being the Tragedy of the Refugees in Russia." By Violetta Thurstan. 2s. 6d. net.

Eyre and Spottiswoode: "A Pilgrimage of the Empire." By Sir Arthur Crosfield, Bart. 1s. net.

Sir Isaac Pitman: "Medical Reporting in Shorthand." By H. Dickinson. 3s. net.

Thacker, Spink and Co., Calcutta: "X-Rays, Electro-Therapeutics, and Radium Therapy." By A. E. Walter, M.R.C.S. 10s. 6d. net.

J. and A. Churchill: "The Story of a Red Cross Unit in Serbia." 6s. net.

**Rates of Subscription**: Twelve months, 6/6; Six months, 4/-; Three months, 2/-, post free to any address in the United Kingdom. Abroad, Twelve months, 8/8; Six months, 5/-; Three months, 2/6, post free. The Hospital "Index" to Volume LIX., October 1915 to March 1916, is now ready, price 1½d. per copy, post free. Address The Manager, "The Hospital," The Hospital Building, 28/29 Southampton Street, Strand, London, W.C.

